# Quantitative assessment of perirenal fat density in renal cell carcinoma: a combined radiologist-driven and YOLO-based CT study

**DOI:** 10.3389/fradi.2026.1911034

**Published:** 2026-10-22

**Authors:** Hadi Sasani, Mazhar Özkan, Mehmet Ali Şimşek, Hacı Murat Akgül, Ayşegül İsal Arslan

**Affiliations:** 1 Department of Radiology, School of Medicine, Tekirdağ Namık Kemal University, Tekirdağ, Türkiye; 2 Department of Software Engineering, Faculty of Engineering and Natural Sciences, Bandırma Onyedi Eylül University, Bandırma, Türkiye

**Keywords:** adipose tissue, computed tomography, Hounsfield units, imaging biomarker, machine learning, perirenal fat density, renal cell carcinoma, YOLOv8

## Abstract

**Aim:**

To compare perirenal adipose tissue density (PRFD) between patients with renal cell carcinoma (RCC) and healthy controls using non-enhanced computed tomography, and to compare manual measurements and YOLOv8-based machine learning (ML) of PRFD.

**Materials and methods:**

The study involved 57 patients with confirmed RCC and 82 control subjects. PRFD was measured manually at different levels of the kidneys and other tumor features. The ML analysis utilized specific attenuation intervals to evaluate PRFD and assess agreement with manual measurements.

**Results:**

RCC group had significantly lower PRFD values at the upper renal pole compared to controls (right: *p* = 0.040; left: *p* = 0.032), while other kidney sections showed no significant differences. In healthy controls, there was a significant right-left difference at all levels, whereas in RCC patients this was seen only at the upper pole (*p* = 0.005). Tumor size was positively correlated with PRFD in specific regions. Tumor volume categories were significantly associated with the presence of tumoral necrosis (*p* = 0.003). The ML method generally aligned with manual measurements, but accuracy varied based on the normalization technique used.

**Conclusion:**

The study found that PRFD varies between RCC patients and controls, particularly at the upper pole, relating more closely to tumor size than to sex or histological type. As the groups were not age-matched, this difference may partly reflect age rather than disease status. The findings suggest that PRFD could be an important imaging marker for RCC evaluation.

## Introduction

Renal cell carcinoma (RCC) represents the most common primary malignancy of the kidney, accounting for approximately 85%–90% of all renal tumors in adults and posing a substantial global health burden due to its rising incidence and heterogeneous biological behavior (1, 2).

Unlike subcutaneous fat, perirenal adipose tissue (PRAT) constitutes a distinct visceral fat depot enclosed by Gerota's fascia and is characterized by rich vascularization, immune cell infiltration, and paracrine activity (3, 4). Experimental and clinical studies suggest that adipose tissue adjacent to solid tumors undergoes metabolic and inflammatory remodeling, which may influence tumor growth, angiogenesis, and local invasion (5–7). However, the radiological manifestations of these changes, particularly in terms of perirenal fat density on computed tomography (CT), remain incompletely understood.

At the cellular and molecular level, RCC-adjacent perirenal fat undergoes a process of cancer-associated adipose tissue remodeling characterized by adipocyte delipidation, increased lipolysis, and a shift toward a pro-inflammatory phenotype. Tumor-derived paracrine signals — including interleukin-6, tumor necrosis factor-alpha, and hypoxia-inducible factor-1*α* — promote the browning of perirenal white adipocytes, upregulate uncoupling protein-1 (UCP1) expression, and stimulate the release of free fatty acids that may fuel tumor metabolic demands (6, 7). Concurrently, inflammatory cell infiltration and extracellular matrix deposition within the peritumoral stroma contribute to fibrotic remodeling of the fat compartment, which is reflected as increased CT attenuation of the adipose tissue (8). The integrity of Gerota's fascia plays a central role in confining these changes: while intact fascia may limit the spatial extent of fat remodeling, its thickening or disruption — detectable on CT — signals more advanced tumor–fat interaction and has been associated with adverse pathological staging in RCC (3, 9).

In addition to tumor size and enhancement characteristics, CT enables objective evaluation of adipose tissue attenuation expressed in Hounsfield units (HU), which reflects underlying tissue composition, including lipid content, fibrosis, and inflammation (8, 10). Prior studies have reported associations between perirenal fat volume or attenuation and adverse oncological features, suggesting that changes in PRAT may serve as indirect imaging markers of tumor–host interaction (9, 11, 12). Nevertheless, reported findings are heterogeneous, and most investigations have relied on limited anatomical sampling or contrast-enhanced protocols, potentially confounding the interpretation of fat density measurements.

In RCC, machine learning (ML) based segmentation models have primarily focused on tumor detection and volumetric assessment, while the application of deep learning to characterize perirenal adipose tissue remains relatively unexplored (13, 14).

The study examined perirenal fat density (PRFD) differences between renal cell carcinoma (RCC) patients and healthy controls using non-enhanced computed tomography (NECT) images. Manual measurements were taken at specific anatomical levels of both kidneys, with a YOLO-based deep learning model used to derive normalized PRFD metrics. The research aimed to combine radiologist measurements with machine learning analysis to explore PRFD changes and assess automated methods for evaluating PRAT in RCC (Supplementary Figure S1) .

## Methods

### Study design and population

This retrospective study analyzed NECT images of patients with histopathologically confirmed renal cell carcinoma (RCC). A total of 118 consecutive RCC patients who were hospitalized between May 20, 2015, and December 27, 2021, were initially screened. Patient ages ranged from 36 to 95 years.

Of these, 61 patients were excluded due to insufficient image quality or the absence of optimum NECT images (motion artifacts, technical limitations, or inadequate image quality).

General inclusion criteria for the study were age ≥20 years and the availability of high-quality NECT images suitable for quantitative analysis. In total, 57 RCC patients constituted the study cohort.

A control group of 82 individuals without known renal or systemic disease was established. Control subjects were selected from healthy individuals aged 30–69 years (mean 50.0 ± 11.9 years). Exclusion criteria for the control group included a history of malignancy, metabolic syndrome, chronic inflammatory or renal disease, trauma and prior renal surgery or intervention.

The control group was evenly distributed by sex (41 men, 41 women) and by age decade (30–39 years, *n* = 20; 40–49 years, *n* = 20; 50–59 years, *n* = 20; 60–69 years, *n* = 22). However, the RCC group was substantially older than the control group (mean 65.1 ± 11.0 vs. 50.0 ± 11.9 years; *p* < 0.001), so the two groups were not formally age-matched despite the balanced internal structure of the control cohort. The potential impact of this age difference on PRFD comparisons is addressed in the Discussion and Limitations.

### Ethical approval

Local ethics committee for non-interventional clinical research approved the study (Approval No: 2022.35.03.03, Date: 29.03.2022). Prior to participation, informed consent was obtained from all patients.

### Scanning protocol and acquisition

CT imaging was performed using a 128-row multi-detector CT scanner (Aquilion™ Prime, Canon Medical Systems) to ensure high-resolution image acquisition. The scanning parameters were optimized to balance image quality and radiation dose, with tube currents modulated based on individual body mass index. The tube current ranged from 100 to 250 mAs, while the tube voltage was set between 100 and 120 kV. Additional parameters included a collimation width of 0.5 mm × 80, a rotation time of 0.35 s, a pitch factor of 0.813, a slice thickness of 1 mm, and a slice interval of 0.8 mm.

### Measurements and assessments

All CT images were independently reviewed by a senior radiologist with 13 years of experience. Image evaluation and measurements were conducted using Sectra 7.0 (Sectra AB, Linköping, Sweden) and Vitrea2® (Vital Images, Minnetonka, MN, USA) workstations to ensure consistency and precision in data extraction.

For RCC cases, tumor characteristics were assessed based on multiple parameters, including laterality (right or left kidney), localization (upper pole, middle part, or lower pole), and orientation (ventral, dorsal, medial, or lateral). Tumor size and volume were recorded. Tumor-associated features such as perilesional streaking and Gerota fascia thickening were documented. To minimize variability, we only included cases imaged with 100–120 kVp and normalized ROI placement across comparable anatomical levels. Standardized circular ROIs (50–70 mm^2^) were manually placed within the perirenal fat, avoiding vessels, fascia, tumor margins, and motion artifacts. Ten ROIs per kidney were analyzed at the upper, middle, and lower poles in axial plane slices (Figure 1).

**Figure 1:**
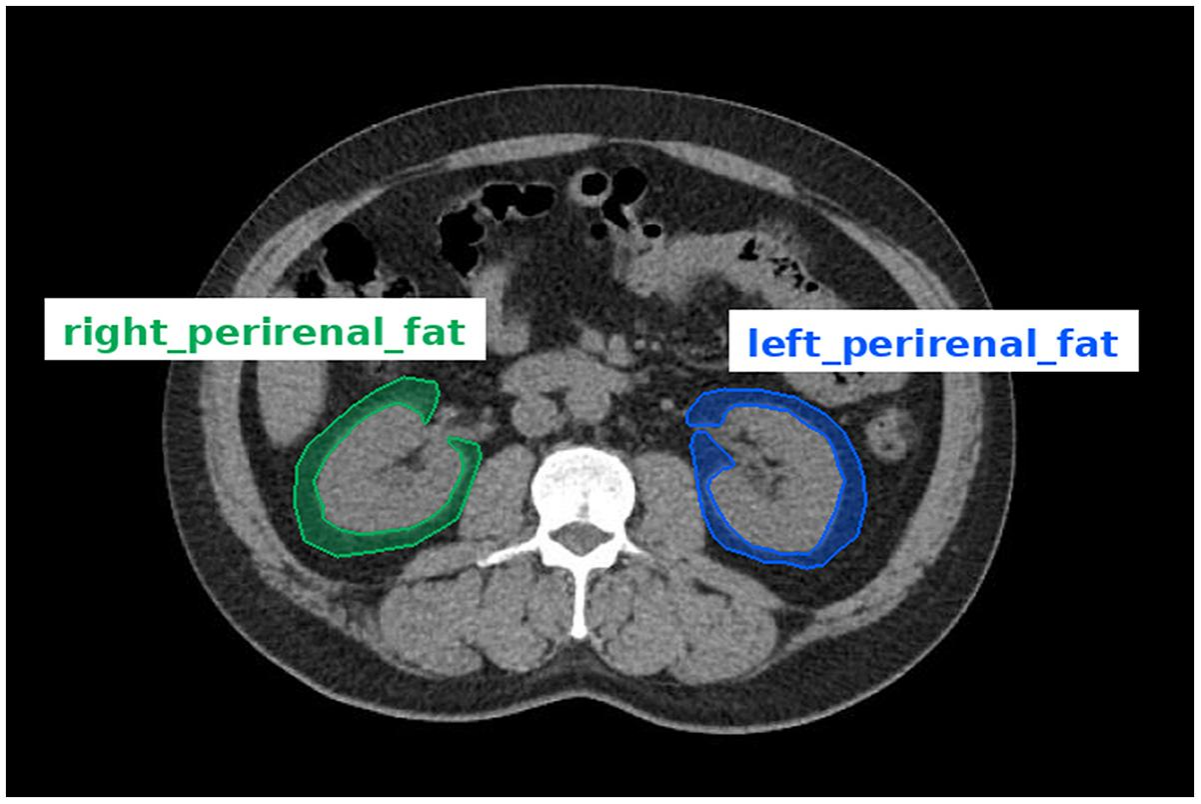
Manual annotation of left (blue) and right (green) perirenal fat tissue surrounding the kidneys.

Comparative analyses of PRFD were conducted using three approaches. First, a horizontal comparison was performed to evaluate PRFD differences between case and control groups at corresponding kidney locations. Second, a vertical comparison analyzed PRFD variations within different regions of the same kidney in RCC patients. Finally, PRFD values were correlated with clinical and pathological parameters, including sex, tumor volume, histopathological diagnosis, and tumor pathological grade, to identify potential associations between perirenal fat characteristics and tumor aggressiveness. The WHO/ISUP (2016) grading system was used for Grade I–IV classification of clear cell and chromophobe RCC, and the WHO/ISUP-endorsed two-tier morphologic classification (Type 1/Type 2) was used for papillary RCC.

### Image segmentation with YOLOv8

CT images were used to segment perirenal adipose tissue using the YOLOv8x-seg model, a high-capacity architecture for object detection and segmentation. All images were resized to 640 × 640 pixels. The model was trained for 100 epochs with a batch size of 2 using stochastic gradient descent (SGD) with a learning rate of 0.01. Training was performed on Google Colab with Python 3.10.12 and Py Torch 2.5.0 + cu121, utilizing an NVIDIA A100 GPU. Both the best and final model weights were saved.

#### Dataset and annotation

The dataset comprised 1,637 CT slices from 139 patients (case and control groups), with ∼12 selected slices per patient, spanning upper, middle, and lower kidney regions. Images were annotated by a senior radiologist and anatomist using two class labels: left_perirenal_fat and right_perirenal_fat. Annotations were performed manually due to subtle anatomical delineation, ensuring high clinical accuracy.

Data were split at the patient level to prevent slice leakage. Segmentation performance was assessed using the Dice coefficient and IoU in addition to mAP50. No external dataset was available, and no k-fold cross-validation was performed—highlighting a need for future external validation. Data was split into training (70%), validation (20%), and testing (10%) sets.

#### Model evaluation

Performance was assessed using precision, recall, and mAP50 metrics. These were computed as follows:

Recall = TP/(TP + FN)
Precisio*n* = TP/(TP + FP)
mAP50 = mean of average precision across all classes at IoU ≥ 0.5

These metrics evaluated detection accuracy and consistency across segmented regions.

#### Post-processing

Segmentation outputs were refined via object-based masking. Each mask was multiplied with the original CT slice to isolate the region of interest (ROI), retaining only perirenal fat tissue and eliminating background structures. This approach enhanced downstream quantitative CT-based assessment by providing anatomically precise, noise-free inputs (Figure 2).

**Figure 2:**
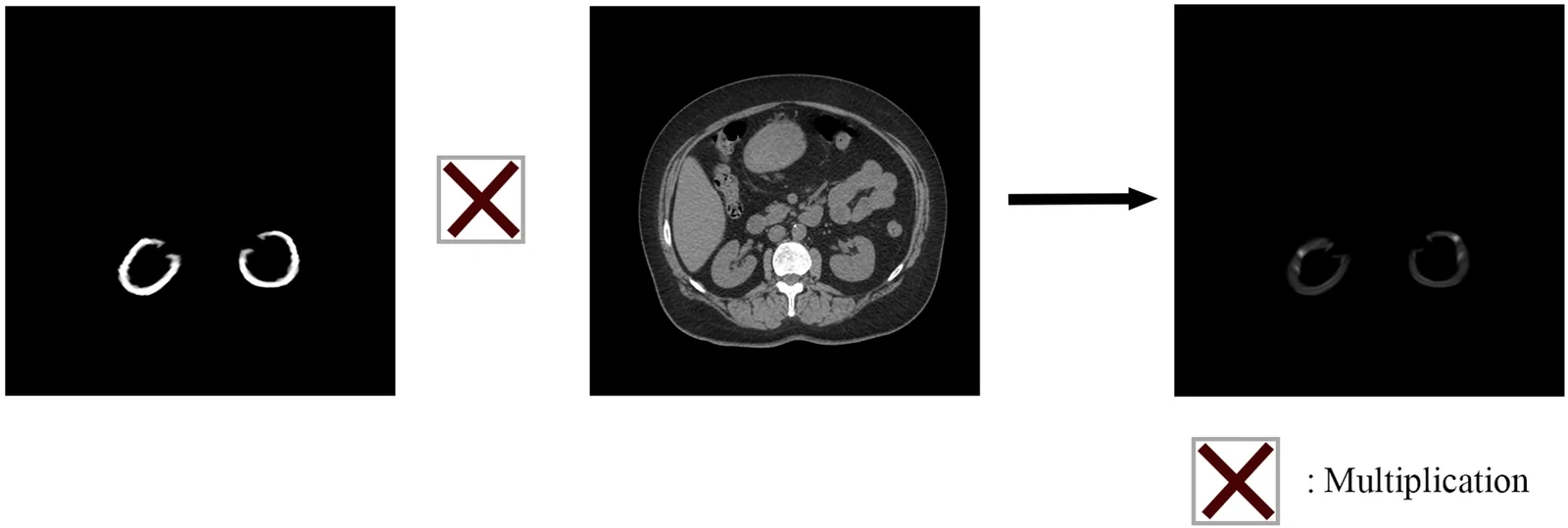
Object-based masking process. The segmentation mask (left) is multiplied with the original image (center) to isolate the target region. As a result (right), the background is removed, and only the classified regions are retained.

### Statistical analysis

Statistical analyses were performed using IBM SPSS Statistics for Windows, version 26.0 (IBM Corp., Armonk, NY, USA). To evaluate data distribution normality tests were performed. Descriptive statistics such as mean, standard deviation, and frequency distributions summarized the dataset. For group comparisons, independent samples t-test and Kruskal–Wallis test were used. Wilcoxon Signed Rank test compared manual and AI measurements. Categorical variables were assessed using the chi-square test. Pearson's correlation was used for normally distributed variables, and Spearman's Rho correlation applied for non-normally distributed variables, with a significance threshold of *p* < 0.05. Area under the ROC curve (AUC) was calculated with 95% confidence intervals using the DeLong method. To classify PRFD measurements by anatomical relationship to the tumor, each of the six measured regions was labeled, per patient, as tumor-adjacent (ipsilateral side, matching pole), ipsilateral non-adjacent, contralateral matched pole, or contralateral non-matched pole, based on recorded tumor side and localization; paired comparisons between these categories used the Wilcoxon signed-rank test. To evaluate potential age-related confounding of case-control PRFD differences, a multivariable linear regression of PRFD on group (case/control), age, and sex was performed *post hoc*.

### AI statement

During the preparation of this work, the authors used QuillBot AI (QuillBot, Version 2, quillbot.com) to check grammar and paraphrase the manuscript. The authors take full responsibility for the accuracy and integrity of all content. This use has also been disclosed in the Acknowledgements section of the title page.

## Results

Comparison of radiological parameters between the case and control groups demonstrated statistically significant differences in PRFD at both the left and right upper renal poles (Table 1).

**Table 1:** Results of measurements related to tumors and PRFD in the patient and control groups.

Parameters	Case (*n* = 57)	Control (*n* = 82)	*p*
Tumor Volume (cc)	139.65 ± 342.45	N/A	N/A
Right Upper Pole PRFD (HU)	−97.64 ± 10.73	−94.99 ± 9.76	**0**.**040**
Right Middle PRFD (HU)	−95.90 ± 10.22	−95.09 ± 8.92	0.225
Right Lower Pole PRFD (HU)	−99.61 ± 11.10	−102.89 ± 7.84	0.092
Left Upper Pole PRFD (HU)	−100.39 ± 10.20	−96.55 ± 10.57	**0**.**032**
Left Middle PRFD (HU)	−95.88 ± 8.12	−97.18 ± 6.51	0.473
Left Lower PRFD (HU)	−98.84 ± 9.32	−101.50 ± 6.19	0.262
Distance of the lesion to the abdominal wall (mm)	38.05 ± 24.36	N/A	N/A

PRFD, perirenal fat density; HU, Hounsfield unit.

Bold values indicate statistical significance (*p* < 0.05).

In the control group, PRFD values differed significantly between the bilateral kidneys across all three renal regions (upper, middle, and lower poles; all *p* < 0.05). In contrast, within the RCC group, a statistically significant right–left difference was observed only at the upper renal pole, while middle and lower pole PRFD values did not differ significantly between sides (Table 2). In side-specific subgroups evaluation, PRFD differences were evident across regions, indicating asymmetrical perirenal fat characteristics, particularly in the presence of renal malignancy.

**Table 2:** Comparison of PRFD values at three different levels in the right and left kidneys in the patient and control groups.

Category	Side	Upper pole PRFD (HU)	Middle part PRFD (HU)	Lower pole PRFD (HU)	*p*
Case (*n* = 57)	Right Side	−97.64 ± 10.73	−95.90 ± 10.22	−99.61 ± 11.10	**0** **.** **002**
	Left Side	−100.39 ± 10.20	−95.88 ± 8.12	−98.84 ± 9.32	**<0** **.** **001**
	*p value*	***0.005***	*0.772*	*0.703*	
Control (*n* = 82)	Right Side	−94.99 ± 9.76	−95.09 ± 8.92	−102.89 ± 7.84	**0** **.** **001**
	Left Side	−96.55 ± 10.57	−97.18 ± 6.51	−101.50 ± 6.19	**<0** **.** **001**
	*p value*	***0.032***	***0.004***	***0.005***	

PRFD, perirenal fat density; HU. Hounsfield unit.

Bold values indicate statistical significance (*p* < 0.05).

No statistically significant differences in PRFD were identified between sexes in both groups (Table 3).

**Table 3:** Comparison of PRFD (perirenal fat density) values at three different levels on the right and left sides according to sex in patient and control groups.

Location	Case		*p*	Control		*p*
	Male (*n* = 39) (mean ± SD)	Female (*n* = 18) (mean ± SD)		Male (*n* = 41) (mean ± SD)	Female (*n* = 41) (mean ± SD)	
Right Upper Pole PRFD (HU)	−96.87 ± 10.97	−99.32 ± 10.29	0.571	−94.51 ± 9.17	−95.47 ± 10.41	0.788
Right Middle PRFD (HU)	−95.22 ± 10.40	−97.39 ± 9.94	0.386	−96.00 ± 6.82	−94.18 ± 10.62	0.510
Right Lower Pole PRFD (HU)	−98.97 ± 11.87	−101.00 ± 9.39	0.618	−102.81 ± 6.11	−102.96 ± 9.33	0.334
Left Upper Pole PRFD (HU)	−99.52 ± 10.86	−102.25 ± 8.59	0.725	−95.51 ± 8.68	−97.59 ± 12.20	0.113
Left Middle PRFD (HU)	−95.74 ± 8.01	−96.18 ± 8.57	0.830	−97.79 ± 5.77	−96.56 ± 7.18	0.280
Left Lower PRFD (HU)	−98.80 ± 9.48	−98.93 ± 9.22	0.744	−101.42 ± 4.65	−101.58 ± 7.49	0.475

PRFD, perirenal fat density; HU, Hounsfield unit; SD, standard deviation.

The most common histopathological type was clear cell carcinoma (*n* = 41, 71.9%) (Table 4). Most lesions were unifocal (98.2%, *n* = 56), while only one case (1.8%) showed multifocality. Additionally, comparison of PRFD values between clear cell renal cell carcinoma (*n* = 41) and papillary renal cell carcinoma (*n* = 12) demonstrated no statistically significant differences across the evaluated renal regions (Table 4). Of the 12 papillary RCC cases, 3 were WHO/ISUP Type 1 and 9 were Type 2; given this small subgroup size, these were analyzed together, and subtype-specific PRFD values are reported descriptively only (see Limitations).

**Table 4:** Comparison of the PRFD values in the two most common RCC groups.

Location	Tumor types		*p*
	Clear cell carcinoma (*n* = 41) (mean ± SD)	Papillary cell carcinoma (*n* = 12) (mean ± SD)	
Right upper pole PRFD (HU)	−97.51 ± 10.21	−98.16 ± 11.67	0.573
Right middle part PRFD (HU)	−96.39 ± 9.52	−95.41 ± 10.94	0.992
Right lower pole PRFD (HU)	−98.91 ± 10.90	−101.06 ± 12.76	0.154
Left upper pole PRFD (HU)	−99.86 ± 11.13	−100.83 ± 6.73	0.882
Left middle part PRFD (HU)	−95.65 ± 7.90	−95.08 ± 9.77	0.932
Left lower part PRFD (HU)	−98.16 ± 9.96	−99.70 ± 6.77	0.831

PRFD, perirenal fat density; HU, Hounsfield unit; SD, standard deviation.

Correlation analyses demonstrated a significant positive association between tumor size parameters and PRFD measured at the left upper renal pole, indicating that increasing tumor dimensions were associated with higher (less negative) PRFD values in this anatomically adjacent region (Table 5).

**Table 5:** Total and side-specific correlations between tumor size parameters and perirenal fat density in renal cell carcinoma patients.

Total	Statistics	Right upper pole PRFD	Right middle pole PRFD	Right lower pole PRFD	Left upper pole PRFD	Left middle pole PRFD	Left lower pole PRFD
AP length	r	.000	.082	−.163	.284*	.228	.158
	p	1.000	.543	.225	**.** **032**	.089	.240
TR length	r	.038	.119	−.106	.369**	.189	.218
	p	.778	.377	.433	.**005**	.158	.103
CC length	r	.011	.110	−.199	.269*	.226	.197
	p	.934	.417	.137	.**043**	.091	.141
Volume	r	−.024	.057	−.163	.221	.204	.137
	p	.859	.674	.226	.099	.127	.311
Right							
AP length	r	.072	.320	−.012	.045	.134	−.054
	p	.717	.097	.950	.819	.497	.783
TR length	r	.180	.400	.050	.146	.202	.106
	p	.358	.**035**	.800	.458	.303	.592
CC length	r	.083	.336	−.071	.021	.151	.004
	p	.674	.080	.721	.916	.443	.982
Volume	r	.080	.327	.057	−.067	.174	.029
	p	.686	.089	.773	.737	.375	.882
Left							
AP length	r	.063	−.050	−.172	.499	.248	.319
	p	.746	.795	.371	.**006**	.195	.091
TR length	r	−.022	−.075	−.210	.543	.146	.357
	p	.911	.700	.275	.**002**	.451	.057
CC length	r	.071	−.058	−.267	.480	.202	.341
	p	.714	.764	.162	.**008**	.293	.071
Volume	r	−.055	−.174	−.310	.434	.144	.240
	p	.778	.367	.102	.**019**	.455	.210

PRFD, perirenal fat density; AP, anteroposterior; TR, transverse; CC, craniocaudal; r, Spearman correlation coefficient.

* *p* < 0.05.

** *p* < 0.01.

Bold values indicate statistical significance (*p* < 0.05).

PRFD values measured at other renal zones demonstrated both positive and negative correlations with tumor size parameters; however, none of these associations reached statistical significance.

In stratification according to tumor laterality, distinct side- and region-specific associations between tumor size and perirenal fat density emerged. Patients with right-sided RCC had a significant positive correlation only between PRFD measured at the right middle renal level and tumor size parameters (r = 0.400, *p* = 0.035). In contrast, patients with left-sided RCC demonstrated weak-to-moderate but more consistent positive correlations between left upper pole PRFD and tumor dimensions as well as tumor volume (r = 0.434, *p* = 0.019). No other renal zones showed statistically significant associations.

Although PRFD differed according to tumor size and localization, no significant associations were observed with tumoral necrosis, Gerota fascia thickening, or histopathological subtype. These findings suggest that PRFD alterations may reflect localized tumor–fat interactions related to spatial tumor expansion rather than downstream pathological features or histological classification. The lack of significance may also be influenced by limited subgroup sizes and the exploratory nature of these analyses, and therefore should be interpreted cautiously.

In stratification of the tumor volume into predefined categories, the presence of tumoral necrosis demonstrated a clear volume-dependent pattern. No tumoral necrosis was observed in patients with tumor volumes less than 17 cc (0/13). In contrast, tumoral necrosis was present in 11 of 20 patients (55.0%) with tumor volumes between 40.01 and 134.00 cc and in 7 of 13 patients (53.8%) with tumor volumes greater than 134 cc. This distribution resulted in a statistically significant association between tumor volume class and tumoral necrosis (Fisher's exact test, *p* = 0.003).

To clarify the anatomical relationship between PRFD alterations and the tumor, each of the six PRFD measurements was classified, per patient, as tumor-adjacent (ipsilateral side, matching pole), ipsilateral non-adjacent (same side, other poles), contralateral matched pole, or contralateral non-matched poles. Tumor-adjacent PRFD (−93.2 ± 11.5 HU) was significantly higher (i.e., less negative, denser) than ipsilateral non-adjacent PRFD (−96.0 ± 16.6 HU; Wilcoxon *p* = 0.0025), contralateral matched-pole PRFD (−100.1 ± 7.7 HU; *p* < 0.0001), and contralateral non-matched-pole PRFD (−99.7 ± 8.4 HU; *p* < 0.0001). This paired, within-patient gradient indicates that PRFD alteration is spatially concentrated at the region directly adjacent to the tumor, supporting a localized tumor–fat interaction rather than a diffuse mechanical-compression effect.

Lymphovascular invasion (LVI) was present in 13/57 RCC cases (22.8%). Comparison of PRFD between LVI-positive and LVI-negative cases did not reach statistical significance at any of the six anatomical regions (*p* = 0.08–0.51), although PRFD was consistently less negative (denser) in the LVI-positive group at every region, a direction consistent with a possible peritumoral inflammatory/fibrotic association that did not reach significance in this modestly sized subgroup.

Since the most significant case-control differences were observed at the upper renal pole, we stratified upper-pole PRFD by tumor location (upper-, middle-, or lower-pole tumors) to test whether this finding was confined to patients whose tumor was itself located at the upper pole. Reduced upper-pole PRFD relative to controls was not restricted to the upper-pole-tumor subgroup (*n* = 19; right *p* = 0.126, left *p* = 0.051): patients with lower-pole tumors (*n* = 17) also showed significantly lower upper-pole PRFD than controls (right *p* = 0.032; left *p* = 0.018), while patients with middle-zone tumors (*n* = 21) did not (right *p* = 0.561, left *p* = 0.870). A Kruskal–Wallis test across the three tumor-location subgroups showed no significant difference in upper-pole PRFD among cases themselves (right *p* = 0.50; left *p* = 0.18). These findings suggest that the upper-pole vulnerability is not simply a local mass effect confined to patients with upper-pole tumors, but may instead reflect a more region-specific, tumor-location-independent phenomenon; however, this observation must be interpreted alongside the age-related confounding discussed below.

Comparison between manual PRFD measurements and AI-based analyses revealed method-dependent differences (Table 6). Significant discrepancies were observed between manual measurements and AI method-1 (−150 to −50 HU) and AI method-2 (−190 to −30 HU) across multiple regions. In contrast, AI method-3 (−125 to +30 HU) showed closer agreement with manual measurements, although statistically significant differences persisted in specific subgroups, including the case group right lower pole (*p* = 0.013), case group left lower pole (*p* = 0.039), case group left upper pole (*p* = 0.021), and control group left upper pole (*p* = 0.012).

**Table 6:** Comparison of manual and machine learning–derived perirenal fat density values across renal levels and sides in renal cell carcinoma patients and controls.

Level	Subgroups	Manual^a^	AI-Method 1^b^ (−150 to −50) HU	AI-Method 2^c^ (−190 to −30) HU	AI-Method 3^d^ (−125 to +30) HU
Right (upper pole)	Case	−97.64 ± 10.73	−132.71 ± 3.39	−162.44 ± 5.41	−96.12 ± 5.14
	Control	−94.99 ± 9.76	−130.16 ± 3.89	−158.38 ± 6.20	−94.36 ± 6.01
Right (middle)	Case	−95.90 ± 10.22	−132.81 ± 3.78	−162.60 ± 6.03	−97.25 ± 5.15
	Control	−95.09 ± 8.92	−131.01 ± 3.56	−159.73 ± 5.68	−95.68 ± 5.50
Right (lower pole)	Case	−99.61 ± 11.10	−133.64 ± 3.08	−163.93 ± 4.90	−96.31 ± 5.93
	Control	−102.89 ± 7.84	−133.22 ± 2.26	−163.25 ± 3.61	−99.09 ± 3.50
Left (upper pole)	Case	−100.39 ± 10.20	−132.29 ± 3.96	−161.77 ± 6.32	−96.61 ± 6.81
	Control	−96.55 ± 10.57	−131.32 ± 3.36	−160.23 ± 5.35	−96.17 ± 5.19
Left (middle)	Case	−95.88 ± 8.12	−132.17 ± 4.33	−161.59 ± 6.90	−95.74 ± 6.63
	Control	−97.18 ± 6.51	−132.15 ± 3.25	−161.55 ± 5.17	−97.44 ± 5.01
Left (lower pole)	Case	−98.84 ± 9.32	−132.76 ± 3.85	−162.49 ± 6.14	−96.49 ± 5.95
	Control	−101.50 ± 6.19	−132.98 ± 2.55	−162.88 ± 4.06	−98.72 ± 3.93

PRFD, perirenal fat density; HU, Hounsfield unit.

ᵃManual ROI-based measurements by the radiologist. ^b,c,d^YOLOv8-derived PRFD values calculated using attenuation intervals of −150 to −50 HU, −190 to −30 HU, and −125 to +30 HU, respectively.

The anatomical localization and spatial distribution of renal tumors according to sex and tumor side are summarized in Supplementary Table S1. Tumors were distributed across the upper, middle, and lower renal poles on both sides, with no clear dominance of a specific pole or quadrant, although dorsal and medial localizations were more frequently observed in lower pole tumors. In both male and female patients, tumors were observed in all renal poles and quadrants, with heterogeneous dorsal, medial, lateral, and ventral distributions across right- and left-sided kidneys, indicating considerable anatomical variability in tumor location.

Comparison of tumor volume categories between male and female patients did not demonstrate statistically significant differences across any volume group; detailed distributions and descriptive statistics are presented in Supplementary Table S2. Tumor volumes were similarly distributed between sexes across all predefined volume categories (<17 cc, 17–40 cc, 40–134 cc, and >134 cc), with no significant sex-related differences in mean tumor volume observed within any category.

Tumor volume distributions according to renal side and anatomical localization (upper, middle, and lower poles) are detailed in Supplementary Table S3. Tumor volumes varied across renal poles on both sides, with larger volumes more frequently observed in upper pole tumors of the left kidney and middle-to-lower pole tumors on the right, reflecting heterogeneity in tumor burden by anatomical location.

Demographic characteristics, tumor volume, lesion-to–abdominal wall distance, and PRFD measurements stratified by histopathological subtype are summarized in Supplementary Table S4. Although clear cell carcinoma constituted most cases, no statistically significant differences in PRFD were observed between clear cell and papillary RCC subtypes across renal levels; findings for chromophobe carcinoma are presented descriptively due to limited case numbers.

Grade-specific distributions of tumor volume, PRFD measurements across renal levels, and lesion-to–abdominal wall distance in clear cell RCC are presented in Supplementary Table S5. Although higher-grade tumors tended to exhibit larger volumes and less negative PRFD values across several renal regions, these findings are presented descriptively due to limited sample sizes within individual grade categories.

The distribution of tumor volume categories according to renal side and type of surgical management (partial vs. radical nephrectomy) is summarized in Supplementary Table S6. Partial nephrectomy was more frequently performed in lower-volume tumors, whereas radical nephrectomy predominated in higher-volume categories on both the right and left sides, with all tumors exceeding 134 cc managed by radical nephrectomy.

Receiver operating characteristic (ROC) analysis demonstrated that perirenal fat density measurements exhibited variable discriminatory performance according to renal side and anatomical level. Upper pole PRFD values on both the right and left sides showed better discrimination between RCC patients and controls compared with middle and lower pole measurements (Figure 3). In contrast, PRFD values measured at the lower poles demonstrated limited discriminatory ability. These findings indicate that the diagnostic performance of PRFD is region-dependent and most pronounced at the upper renal pole. The corresponding AUC values (with 95% confidence intervals, DeLong method) were: right upper pole, 0.603 (0.505–0.700); right middle, 0.561 (0.462–0.659); right lower pole, 0.416 (0.319–0.513); left upper pole, 0.607 (0.511–0.704); left middle, 0.464 (0.361–0.567); left lower pole, 0.444 (0.344–0.544). Only the upper-pole regions achieved an AUC significantly greater than 0.5, and these values indicate modest rather than strong discriminatory performance.

**Figure 3:**
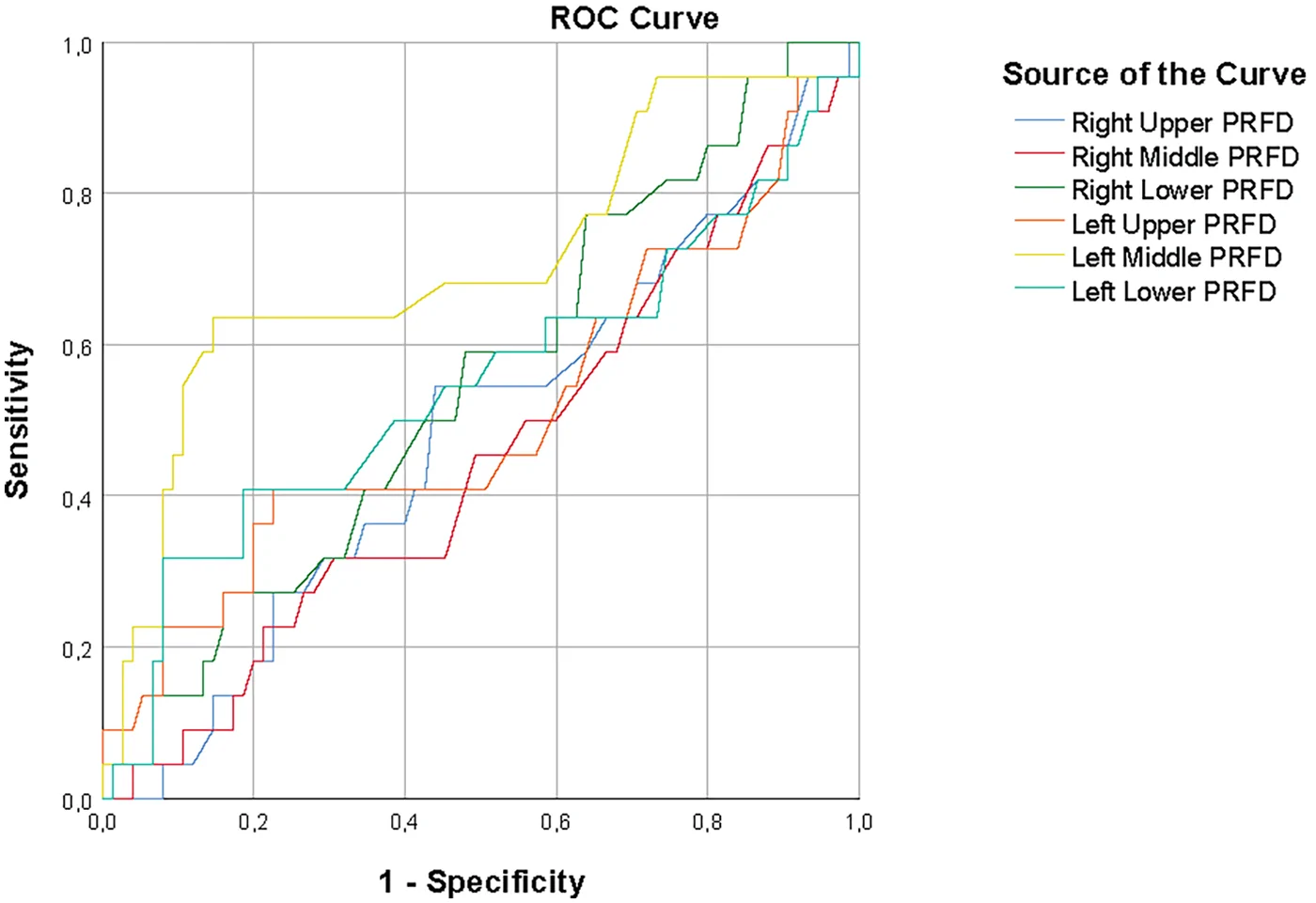
Receiver operating characteristic curves for right and left perirenal fat density measurements.

### Machine learning and segmentation

The segmentation performance of the YOLOv8-seg model was evaluated using segmentation-specific metrics, including Precision, Recall, and Average Precision at IoU threshold 0.50 (mAP50). Precision reached 79.6% at the best epoch and 75.5% at the final epoch, indicating that the model accurately delineated object boundaries and achieved the desired segmentation specificity. Recall values were observed at 78.8% (best epoch) and 78.7% (final epoch), reflecting the model's consistent ability to capture target objects comprehensively with correct pixel matches.

The mAP50 values were 82.98% at the best epoch and 79.61% at the final epoch, demonstrating the model's stable segmentation accuracy and reliable mask predictions across different data samples. These outcomes indicate that the YOLOv8-seg model delivered effective and goal-oriented performance in the segmentation task.

All performance metrics—Precision, Recall, and mAP50—exhibited a steady increase during training and plateaued at high values without signs of overfitting. Over the course of 100 epochs, the metrics consistently reached high levels of accuracy, confirming the model's robust learning capability and stable generalization across the dataset (Figure 4).

**Figure 4:**
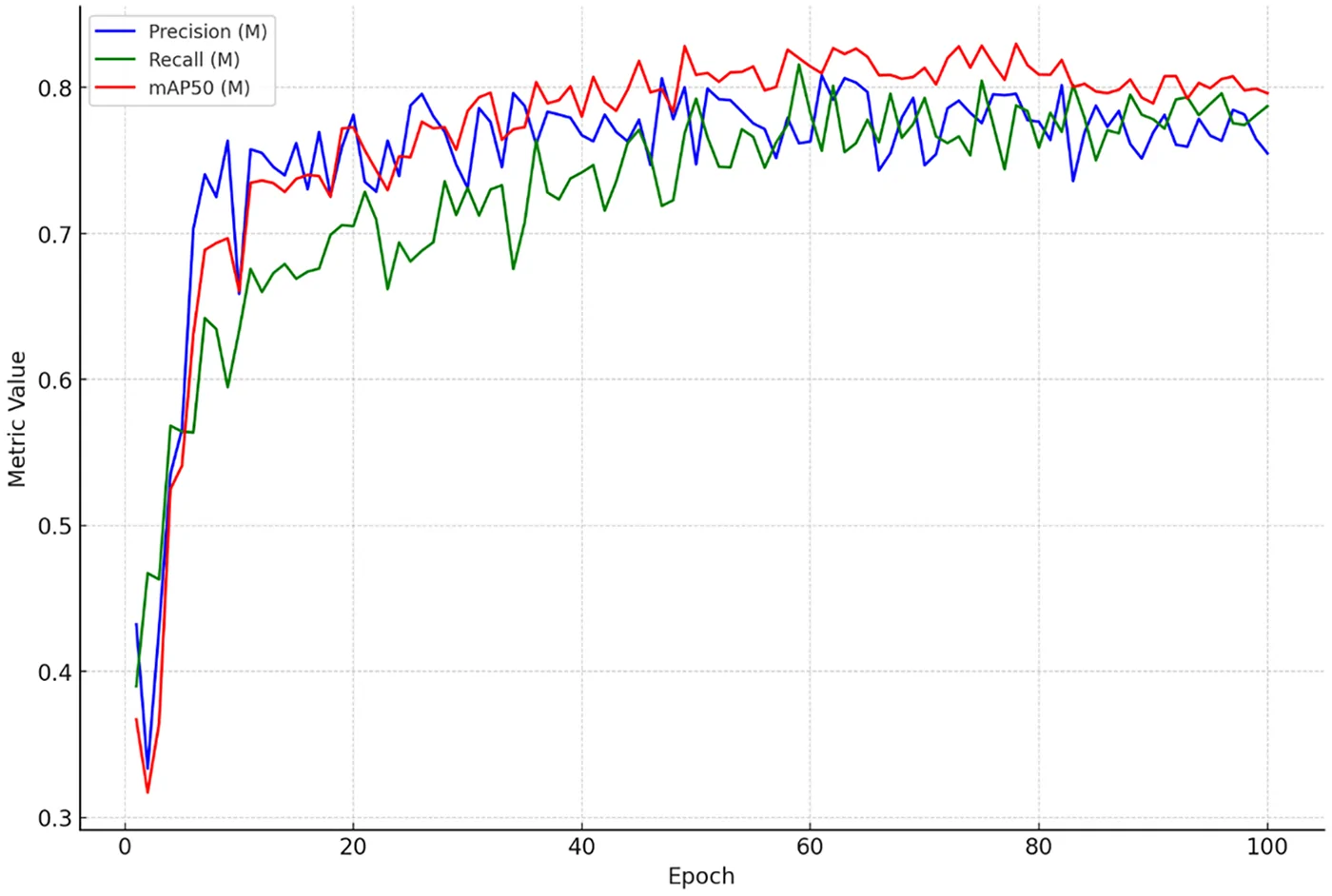
Performance metric trends (Precision, Recall, mAP50) throughout the training of the YOLOv8-seg model.

To assess the model's generalization capability, a test dataset comprising previously unseen samples with characteristics like the training data was used. Examples from the test dataset are shown in Figure 5 to show how well the model segments new cases.

**Figure 5:**
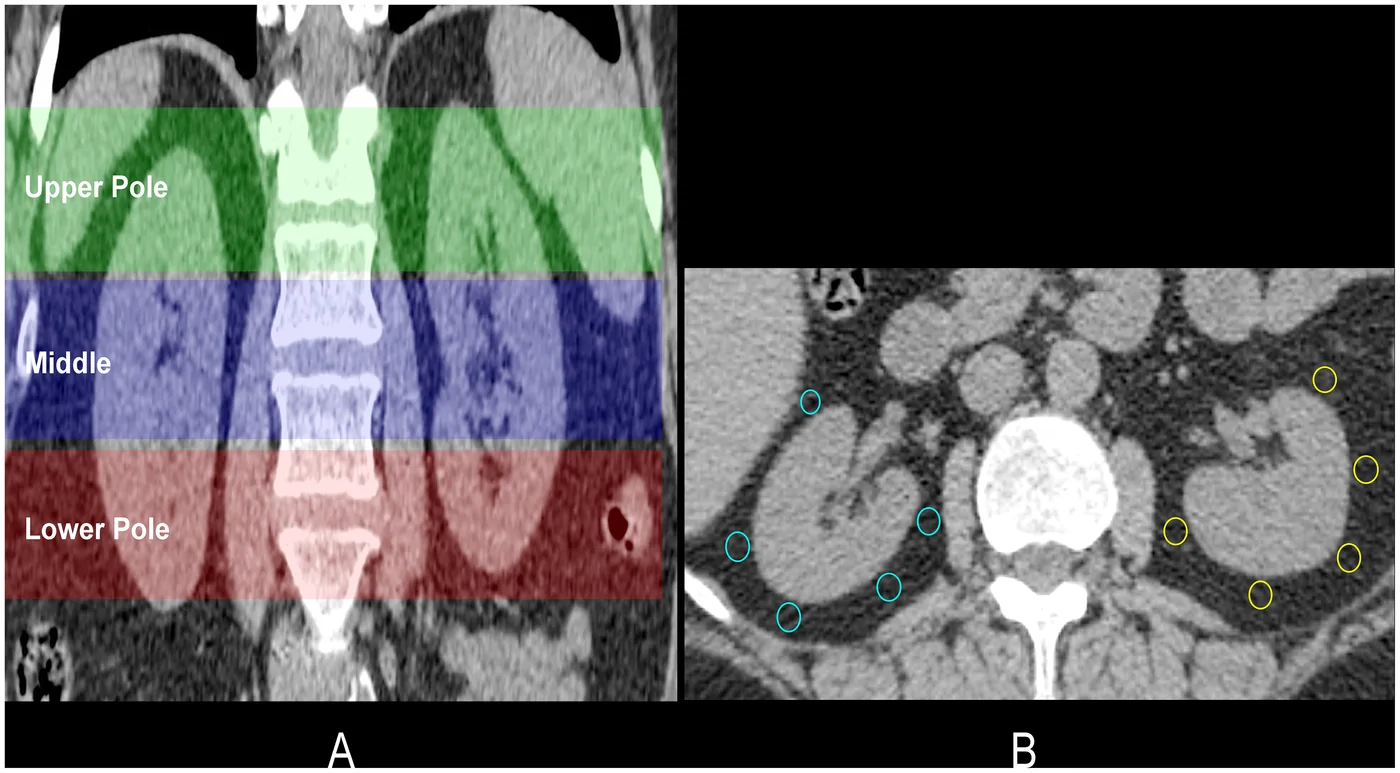
Examples from the test dataset, demonstrating the model's segmentation performance.

In the analysis of data segmented using machine learning, statistically significant differences were observed when compared to the results obtained through manual methods. ML-derived PRFD values showed lower standard deviations than manual measurements, indicating less inter-individual variability. Table 6 compares PRFD values generated through machine learning segmentation and manual labeling across anatomical localizations in cancer (case) and non-cancer (control) groups. With AI method-1 (−150 to −50 HU), the most pronounced intergroup differences were observed at the right upper pole and right middle region, where ML-derived PRFD values were lower in cancer patients than in controls (right upper pole: −132.71 ± 3.39 HU vs. −130.16 ± 3.89 HU, *p* < 0.001; right middle: −132.81 ± 3.78 HU vs. −131.01 ± 3.56 HU, *p* = 0.006). A similar pattern was observed with AI method-2 (−190 to −30 HU) at the same locations (right upper pole: −162.44 ± 5.41 HU vs. −158.38 ± 6.20 HU, *p* < 0.001; right middle: −162.60 ± 6.03 HU vs. −159.73 ± 5.68 HU, *p* = 0.006).

## Discussion

The present study demonstrates that PRFD measured on NECT differs between RCC patients and healthy controls in a side- and level-dependent manner. Specifically, RCC patients exhibited significantly lower PRFD values at the upper renal poles bilaterally, while no significant differences were observed at the middle or lower poles in pooled analyses. These findings reinforce the concept that perirenal adipose tissue represents an active and locally responsive component of the renal tumor microenvironment rather than a passive anatomical structure (15, 16).

Side-specific analyses further revealed physiological asymmetry in perirenal fat density among control subjects, with significant right–left differences across all renal regions. This observation is consistent with known anatomical and functional asymmetries related to renal position, adjacent organs, and vascular architecture (17). In contrast, RCC patients demonstrated significant side-related differences only at the upper pole, suggesting that tumor presence may partially disrupt baseline perirenal fat distribution patterns in a localized manner. These findings highlight the importance of region- and side-specific evaluation when assessing PRFD and suggest that global or average measurements may obscure biologically relevant local changes.

Tumor size analyses provided additional insight into the spatial relationship between RCC and adjacent adipose tissue. Significant positive correlations were observed between tumor size parameters and PRFD measured at the ipsilateral upper pole, particularly in left-sided tumors. This pattern suggests that linear tumor expansion toward the perirenal fat compartment may influence local fat composition more directly than overall tumor volume. Similar localization-dependent tumor–fat interactions have been described in prior CT-based studies evaluating peritumoral adipose tissue in solid organ malignancies, including RCC. A direct, within-patient comparison classifying each PRFD measurement by its anatomical relationship to the tumor supported this interpretation: PRFD at the tumor-adjacent pole was significantly higher (denser) than at the ipsilateral non-adjacent poles, the contralateral matched pole, and the contralateral non-matched poles, indicating that the alteration is spatially concentrated rather than diffuse. Nevertheless, when upper-pole PRFD was stratified by tumor location, reduced values relative to controls were also present in patients whose tumor was located away from the upper pole and did not differ significantly among tumor-location subgroups within the RCC group. Taken together, these findings suggest that PRFD alterations combine a genuine, spatially localized component adjacent to the tumor with a broader, region-specific (upper-pole) susceptibility that is not fully explained by tumor position alone (18).

The AUC values for case-control discrimination were modest (0.60–0.61 at the upper poles, not significantly different from chance elsewhere), indicating that PRFD alone has limited standalone diagnostic performance and should be interpreted as a supportive, rather than primary, imaging marker. A clinically more relevant application would be discriminating benign from malignant renal masses in patients with indeterminate lesions, which could help reduce unnecessary surgical intervention; however, the present case-control design, which compared confirmed RCC with healthy controls rather than a cohort of pathologically confirmed benign renal masses, cannot directly test this question. The modest AUC values reported here provide preliminary, hypothesis-generating support for this application, which should be evaluated directly in future prospective studies incorporating indeterminate and benign renal masses (8, 18).

Although PRFD did not differ according to sex or between clear cell and papillary RCC subtypes, tumor volume categories were strongly associated with the presence of tumoral necrosis. Necrosis was absent in small-volume tumors and increasingly prevalent in higher-volume groups, consistent with established pathological mechanisms linking tumor burden, hypoxia, and necrotic transformation (2). Importantly, the absence of a consistent association between PRFD and necrosis suggests that perirenal fat alterations may primarily reflect localized tumor–fat interface effects rather than secondary pathological changes associated with advanced disease.

Comparison of manual PRFD measurements with YOLOv8-based machine learning (ML) analysis demonstrated that automated segmentation yields PRFD distributions broadly consistent with radiologist-driven assessment, while also revealing method-dependent differences related to attenuation normalization strategy. The closest agreement between manual and AI-derived measurements was observed using the −125 to +30 HU interval, whereas wider attenuation ranges resulted in greater divergence. These findings emphasize that the performance of AI-based fat analysis depends critically on appropriate attenuation threshold selection and underscore the importance of standardized imaging biomarker definitions, as highlighted by recent radiomics and imaging biomarker standardization initiatives (14, 19).

From a biological perspective, localized increases in PRFD likely reflect a combination of low-grade inflammation, fibrosis, and microvascular remodeling induced by tumor-derived paracrine signaling. Experimental and clinical studies have shown that adipose tissue adjacent to solid tumors undergoes metabolic and structural changes that increase CT attenuation, even in the absence of overt invasion (8, 10). Hypoxia-driven angiogenesis and extracellular matrix deposition further contribute to these alterations, supporting the mechanistic plausibility of PRFD as a marker of localized tumor–fat interaction (20).

Several limitations of this study should be acknowledged. Most importantly, the RCC and control groups differed substantially in age (mean 65.1 ± 11.0 vs. 50.0 ± 11.9 years; *p* < 0.001), despite the internally balanced age-decade structure of the control cohort; the groups were therefore not truly age-matched. In an exploratory multivariable linear regression of upper-pole PRFD on group, age, and sex, the case-control difference was no longer statistically significant after adjusting for age (*p* = 0.51 right, *p* = 0.56 left), whereas age itself was a strong, independent, inverse predictor of PRFD (*p* < 0.001 at both sides; within controls alone, age correlated with right upper-pole PRFD at r = −0.535, *p* < 0.001, a correlation not present within cases, r = −0.038, *p* = 0.78). This finding indicates that age-related confounding cannot be excluded as a contributor to the observed upper-pole PRFD difference, and the main case-control finding of this study should accordingly be interpreted with caution pending confirmation in a properly age-matched cohort with adequately powered multivariable modeling incorporating tumor size, necrosis, and lymphovascular invasion. In addition, exclusion of overt metabolic syndrome or inflammatory disease in controls does not preclude subclinical individual variation in perirenal adipose tissue composition and inflammatory status, which may have contributed unexplained variance to control-group PRFD measurements. Papillary RCC Type 1 and Type 2 tumors (*n* = 3 and *n* = 9, respectively) were analyzed together because of insufficient sample size for a reliable subgroup comparison, which may obscure biologically relevant differences between these subtypes, and the lymphovascular invasion subgroup (*n* = 13) was similarly small, limiting the power to detect a true association with PRFD. The retrospective, single-center design and modest sample size limited the statistical power of subgroup analyses, particularly for less common histological subtypes. Additionally, although ML-based segmentation improved reproducibility, partial reliance on manual region selection was required for validation. Future studies should focus on external validation in larger, age-matched cohorts, the development of fully automated segmentation pipelines, and the integration of multimodal imaging or molecular data to further elucidate the biological and clinical significance of perirenal fat alterations in RCC (10, 13).

## Conclusion

This study demonstrates that PRFD on NECT differs between RCC patients and healthy controls in a side- and region-specific manner, with the most consistent differences observed at the upper renal poles. PRFD alterations were spatially heterogeneous and showed localized associations with tumor size parameters, particularly in anatomically adjacent regions, while no significant differences were identified according to sex or between clear cell and papillary histological subtypes. However, because the RCC and control groups were not age-matched and the upper-pole difference did not persist after adjustment for age, this finding should be interpreted cautiously and confirmed in an age-matched cohort.

Within-group analyses revealed physiological right–left asymmetry in PRFD among control subjects, whereas this pattern was partially attenuated in RCC patients, suggesting localized disruption of baseline perirenal fat distribution in the presence of malignancy. Tumor volume was strongly associated with the presence of tumoral necrosis and surgical management approach, confirming expected relationships between tumor burden and pathological and clinical features.

Machine learning–assisted segmentation using a YOLO-based model provided PRFD measurements broadly consistent with manual assessment, with agreement dependent on the selected attenuation normalization interval. These findings highlight the potential utility of automated approaches for standardized and reproducible evaluation of perirenal fat, while emphasizing the importance of appropriate parameter selection.

Overall, PRFD reflects localized tumor–fat interactions rather than global disease characteristics and may serve as a supportive imaging marker in the evaluation of RCC. Further validation in larger, age-matched, multicenter cohorts is warranted to clarify its diagnostic value and to optimize the integration of automated PRFD assessment into clinical workflows.

## Data Availability

The raw data supporting the conclusions of this article will be made available by the authors, without undue reservation.
