# Supplementary material for: Quantitative assessment of perirenal fat density in renal cell carcinoma: a combined radiologist-driven and YOLO-based CT study
Source: Front Radiol. 2026 Oct 22;6:1911034. doi: 10.3389/fradi.2026.1911034 (PMC13736697; doi:10.3389/fradi.2026.1911034)
Supplement: Supplementary file 1 [file Table1.docx]

**Supplementary Table 1. Tumor level and orientation according to sex and side.**

| **Sex** | **Tumor side** | **Renal level** | **n (% within sex)** | **Orientation distribution within cell** |
| --- | --- | --- | --- | --- |
| Male | Right | Upper pole | 7 (17.9%) | Dorsal: 0 (0.0%); Ventral: 3 (42.9%); Lateral: 1 (14.3%); Medial: 3 (42.9%) |
| Male | Right | Middle zone | 7 (17.9%) | Dorsal: 1 (14.3%); Ventral: 2 (28.6%); Lateral: 0 (0.0%); Medial: 4 (57.1%) |
| Male | Right | Lower pole | 6 (15.4%) | Dorsal: 4 (66.7%); Ventral: 0 (0.0%); Lateral: 1 (16.7%); Medial: 1 (16.7%) |
| Male | Left | Upper pole | 7 (17.9%) | Dorsal: 2 (28.6%); Ventral: 4 (57.1%); Lateral: 0 (0.0%); Medial: 1 (14.3%) |
| Male | Left | Middle zone | 6 (15.4%) | Dorsal: 2 (33.3%); Ventral: 0 (0.0%); Lateral: 3 (50.0%); Medial: 1 (16.7%) |
| Male | Left | Lower pole | 6 (15.4%) | Dorsal: 2 (33.3%); Ventral: 1 (16.7%); Lateral: 0 (0.0%); Medial: 3 (50.0%) |
| Female | Right | Upper pole | 3 (16.7%) | Dorsal: 1 (33.3%); Ventral: 1 (33.3%); Lateral: 0 (0.0%); Medial: 1 (33.3%) |
| Female | Right | Middle zone | 3 (16.7%) | Dorsal: 1 (33.3%); Ventral: 0 (0.0%); Lateral: 1 (33.3%); Medial: 1 (33.3%) |
| Female | Right | Lower pole | 2 (11.1%) | Dorsal: 2 (100.0%); Ventral: 0 (0.0%); Lateral: 0 (0.0%); Medial: 0 (0.0%) |
| Female | Left | Upper pole | 2 (11.1%) | Dorsal: 0 (0.0%); Ventral: 1 (50.0%); Lateral: 0 (0.0%); Medial: 1 (50.0%) |
| Female | Left | Middle zone | 5 (27.8%) | Dorsal: 1 (20.0%); Ventral: 1 (20.0%); Lateral: 3 (60.0%); Medial: 0 (0.0%) |
| Female | Left | Lower pole | 3 (16.7%) | Dorsal: 3 (100.0%); Ventral: 0 (0.0%); Lateral: 0 (0.0%); Medial: 0 (0.0%) |

**Supplementary Table 2. Tumor volume categories according to sex.**

| **Volume category, cm3** | **Male (n=39), n (%); mean ± SD** | **Female (n=18), n (%); mean ± SD** | **p** |
| --- | --- | --- | --- |
| <17 | 8 (20.5%); 9.89 ± 5.06 | 5 (27.8%); 9.21 ± 3.76 | 0.724 |
| 17-40 | 7 (17.9%); 26.10 ± 7.13 | 4 (22.2%); 30.04 ± 1.85 | 0.109 |
| 40.01-134 | 16 (41.0%); 76.67 ± 24.79 | 4 (22.2%); 80.70 ± 25.52 | 0.820 |
| >134 | 8 (20.5%); 534.67 ± 764.47 | 5 (27.8%); 341.04 ± 380.20 | 0.943 |

*p values compare tumor volume between sexes within each category using the Mann-Whitney U test.*

**Supplementary Table 3. Tumor volume according to side, renal level, and volume category.**

| **Side** | **Renal level** | **<17** | **17-40** | **40.01-134** | **>134** |
| --- | --- | --- | --- | --- | --- |
| Right | Upper pole | 12.06 ± 2.52 (n=5) | 32.58 (n=1) | 76.24 ± 34.20 (n=3) | 141.94 (n=1) |
| Right | Middle zone | 8.98 ± 0.76 (n=2) | 28.30 ± 15.37 (n=2) | 68.21 ± 20.56 (n=3) | 189.51 ± 51.95 (n=3) |
| Right | Lower pole | - | 28.11 (n=1) | 70.24 ± 14.25 (n=7) | - |
| Left | Upper pole | - | 27.87 ± 0.70 (n=3) | 94.47 ± 15.96 (n=2) | 1098.67 ± 898.04 (n=4) |
| Left | Middle zone | 7.59 ± 5.64 (n=4) | 26.47 ± 5.57 (n=3) | 84.96 ± 32.44 (n=3) | 163.27 (n=1) |
| Left | Lower pole | 8.29 ± 8.08 (n=2) | 22.55 (n=1) | 90.34 ± 52.50 (n=2) | 178.52 ± 28.22 (n=4) |

*Values are mean ± standard deviation in cm^3^ unless n=1.*

**Supplementary Table 4. Demographic, tumor, and regional PRFD values according to histological subtype.**

| **Parameter** | **Clear cell RCC (n=41)** | **Papillary RCC (n=12)** | **Chromophobe RCC (n=4)** |
| --- | --- | --- | --- |
| Age, years | 64.85 ± 10.56 | 66.67 ± 12.76 | 63.50 ± 12.40 |
| Tumor volume, cm3 | 108.52 ± 205.63 | 249.75 ± 650.76 | 128.46 ± 103.02 |
| Right upper pole PRFD, HU | -97.51 ± 10.21 | -98.16 ± 11.67 | -97.50 ± 16.07 |
| Right middle level PRFD, HU | -96.39 ± 9.52 | -95.41 ± 10.94 | -92.37 ± 16.79 |
| Right lower pole PRFD, HU | -98.91 ± 10.90 | -101.06 ± 12.76 | -102.46 ± 9.69 |
| Left upper pole PRFD, HU | -99.86 ± 11.13 | -100.83 ± 6.73 | -104.46 ± 9.90 |
| Left middle level PRFD, HU | -95.65 ± 7.90 | -95.08 ± 9.77 | -100.61 ± 3.66 |
| Left lower pole PRFD, HU | -98.16 ± 9.96 | -99.70 ± 6.77 | -103.24 ± 9.77 |
| Lesion-to-abdominal-wall distance, mm | 35.21 ± 22.92 | 41.37 ± 22.22 | 57.25 ± 40.21 |

*The six regional measurements are fixed anatomical renal levels and are not necessarily in direct contact with the tumor. The tumor-level-specific ipsilateral analysis is presented in main Table 3. Papillary RCC comprised Type I (n=3) and Type II (n=9).*

**Supplementary Table 5. Tumor and PRFD values according to Fuhrman grade in clear cell RCC.**

| **Parameter** | **Grade I (n=18)** | **Grade II (n=10)** | **Grade III (n=7)** | **Grade IV (n=6)** |
| --- | --- | --- | --- | --- |
| Age, years | 64.67 ± 8.06 | 60.90 ± 12.31 | 67.86 ± 5.58 | 68.50 ± 17.34 |
| Tumor volume, cm3 | 32.64 ± 34.63 | 66.08 ± 47.56 | 244.20 ± 345.77 | 248.59 ± 336.17 |
| Right upper pole PRFD, HU | -99.25 ± 8.50 | -99.97 ± 6.31 | -94.99 ± 16.02 | -91.12 ± 11.26 |
| Right middle level PRFD, HU | -100.16 ± 6.09 | -96.56 ± 5.84 | -96.13 ± 10.15 | -85.14 ± 14.53 |
| Right lower pole PRFD, HU | -102.75 ± 5.81 | -99.61 ± 7.13 | -96.71 ± 16.82 | -88.79 ± 14.87 |
| Left upper pole PRFD, HU | -105.27 ± 6.49 | -99.13 ± 8.02 | -92.90 ± 15.66 | -92.96 ± 14.35 |
| Left middle level PRFD, HU | -99.52 ± 5.60 | -94.77 ± 7.62 | -92.92 ± 8.61 | -88.66 ± 8.66 |
| Left lower pole PRFD, HU | -102.10 ± 4.78 | -97.57 ± 7.84 | -92.56 ± 17.21 | -93.86 ± 11.29 |
| Lesion-to-abdominal-wall distance, mm | 34.57 ± 18.63 | 37.87 ± 27.72 | 36.54 ± 33.07 | 31.17 ± 17.03 |

*WHO/ISUP nuclear grade was applied to clear cell RCC. Values are mean ± standard deviation.*

**Supplementary Table 6. Tumor volume categories according to side and surgical procedure.**

| **Side** | **Surgical procedure** | **<17** | **17-40** | **40.01-134** | **>134** |
| --- | --- | --- | --- | --- | --- |
| Right | Partial nephrectomy | 6 (85.7%) | 1 (25.0%) | 4 (30.8%) | 0 (0.0%) |
| Right | Radical nephrectomy | 1 (14.3%) | 3 (75.0%) | 9 (69.2%) | 4 (100.0%) |
| Left | Partial nephrectomy | 6 (100.0%) | 4 (57.1%) | 1 (14.3%) | 0 (0.0%) |
| Left | Radical nephrectomy | 0 (0.0%) | 3 (42.9%) | 6 (85.7%) | 9 (100.0%) |

*Percentages are calculated within each side and tumor-volume category.*
